# Stability evaluation of open-pit mine slope based on Bayesian optimization 1D-CNN

**DOI:** 10.1038/s41598-024-64663-8

**Published:** 2024-06-18

**Authors:** Jinguo Lyu, Taihong Hu, Guangwei Liu, Bo Cao, Wenqi Wang, Shixu Li

**Affiliations:** 1 College of Mining, Liaoning Technical University, Fuxin, 123000 China; 2 School of Mechanics and Engineering, Liaoning Technical University, Fuxin, 123000 China

**Keywords:** Open-pit mines, Slope stability, Bayesian optimization, Natural hazards, Computational science

## Abstract

As mechanized open-pit coal mining intensifies, assessing and predicting slope stability has become increasingly important. To address the limitations of traditional mechanical calculations, numerical simulations, and physical experiments, this paper identifies the key factors impacting slope stability in open-pit mines and develops a multi-parameter sample data set. The study employs hyperparameters optimized using a Bayesian algorithm, introduces additional convolutional layers, and combines the Adam optimizer with dropout techniques to enhance the feature extraction and performance of one-dimensional convolutional neural networks (1D-CNN). This leads to a Bayesian-optimized one-dimensional convolutional neural network (B-1D MCNN) model for predicting slope stability.The study evaluates the classification performance and accuracy of various models for slope stability, including BP neural networks, genetic algorithm-optimized convolutional neural networks, 1D-CNN, and B-1D MCNN, using accuracy, precision, and F1-score as metrics. The analysis also examines the influence of factor indicators and training set length on the model's output to assess its generalization capabilities.The research findings suggest that: (1) the B-1D MCNN model for evaluating slope stability demonstrates the capability to accurately depict the nonlinear correlation between influencing factors and slope stability. (2) Compared with other models, the B-1D MCNN model has shown enhancements of 10.96% to 27.85%, 10.26% to 28.55%, and 8.98% to 25.05% in terms of Accuracy, F1-Score, and Precision, respectively. (3) As the length of the training dataset increases, the performance of the model improves accordingly. (4) The B-1D MCNN model shows a generalization power of 87.5%.

## Introduction

In China, large-scale and high-intensity open-pit coal mining has resulted in increasingly serious slope stability issues. For example, on February 22, 2023, the Inner Mongolia Alashan Xinjing Coal Co., Ltd. open-pit coal mine suffered a very severe slope instability and collapse accident, killing 53 people and injuring 6. Obviously, the evaluation and prediction of slope stability in open pit mines has become a hot and difficult problem that needs to be developed and improved^1–3^.

There are currently three main methods of slope stability research in use in the industry, which have significant implications for engineering design: rigid body limit analysis, numerical simulation analysis and physical similarity model experimentation. The rigid-body limit equilibrium method is a traditional mechanical analysis methodology that assesses slope stability. It estimates the relationship between anti-slip force and sliding force on the slope's potential sliding body. This approach is easy to compute and frequently utilized in the industry. However, the method needs the selection of a typical engineering geological section, assumes the sliding mode and location of the sliding surface in advance, and does not take into account the sliding body's deformation damage. This approach solely solves the sliding problem of the sliding body, which causes the slope stability coefficient estimate to deviate slightly from the actual scenario^4,5^.The numerical simulation calculation method has become widely used with the development of computer science. A three-dimensional slope finite element visualization model is established to determine the physical and mechanical parameters of the rock and soil layer. The appropriate constitutive model is selected, and boundary conditions are applied. Through a large number of iterative calculations, the deformation and damage inside the slope are obtained. The method provides an intuitive and robust way to obtain information on the overall and local deformation and destruction of the evolution process. However, it is challenging to consider the non-homogeneity and anisotropy of the geotechnical layer. Additionally, the calculation time is lengthy, and the results are significantly affected by the constitutive model used, with varying results observed for different models^6,7^.The physical similarity experiment approach, which is based on an actual engineering background, takes into account the similarity ratio of rock and soil materials, geometric dimensions, and material strength. The physical similarity model of an open-pit slope is designed to more accurately depict the deformation, damage, and movement of slope rock and soil throughout the mining operation. However, the classic experiment is linked to a physical object, whereas the geological conditions of the slope in the actual project are more complex. When the study item is complex and difficult to operate, it will require a significant amount of labor and material resources, but it will not ensure the smooth completion of the experiment.

The three methods mentioned above are widely used in engineering practice. In recent years, other analysis methods have been proposed and developed, such as mathematical statistics, expert assessment, fuzzy comprehensive evaluation, regression analysis and artificial intelligence analysis. Although these methods have been applied to some extent, Artificial intelligence analysis^8^ is a popular method that is strongly advocated by the state and is developing at a rapid pace. It uses computers as a platform, applies mathematical algorithms, and builds intelligent perception models. It has the ability to learn from experience, particularly from mistakes, and can be developed and perfected by learning from a large number of cases^9–11^.Given the abundance of classic slope engineering cases, slope stability evaluation and prediction can be achieved through continuous learning. This approach avoids the drawbacks of complex model construction and non-homogeneous mechanical analysis.

Neural networks are a widely used and reliable method of artificial intelligence^12–18^. The method has been used in slope engineering research^19–22^ as a versatile and potentially adaptive way to developing effective slope stability prediction models for nonlinear complicated issues. It is less reliant on assumptions and more objective than prior approaches. The slope stability assessment was successfully carried out using the BP neural network algorithm and the GA-BP optimized neural network, as reported in references^23–25^.The backpropagation (BP) network algorithm has clear advantages in solving nonlinear problems. However, it is based on the gradient method, which results in slow convergence, susceptibility to overfitting, and local minima.

Convolutional Neural Network (CNN)^26^ surpasses the BP algorithm's limitations with its local connections, weight sharing, and strong adaptive feature extraction. It effectively handles complex sample data, reducing network model complexity while enhancing feature extraction and computational abilities. CNNs have been applied in predicting stability on the northern slopes of the Himalayan mountain area^27^, analyzing the nonlinear relationship between open-pit mine slope stability and influencing factors^28^, and studying the impact of CNN network model parameters on slope stability prediction. However, CNN hyperparameters are usually tuned using empirical, cross-validation, and experimental methods in the application process, and there is a lack of optimization-seeking criteria to select the best hyperparameter combinations^29^, and the constructed CNN network model still has the potential to be improved in slope stability prediction.

In the current work the main effective factors on the slope stability to train the suggested One-dimensional convolutional neural network model (1D-CNN) were selected in accordance with the previous studies^30–33^. Based on this, the Bayesian algorithm is used to optimize the hyperparameters of the 1D-CNN model. The model's feature learning capacity is improved through adding convolutional layers, using the ReLU activation function, and combining the Adam optimizer and dropout approach^34–36^.

## Optimized 1D-CNN model

### Principle of convolutional neural network

A convolutional layer, an output layer, a pooling layer, a fully connected layer, and an input layer typically make up a convolutional neural network structure. The convolution and pooling layers carry out feature extraction and dimensionality reduction after the input layer has processed the input data. Subsequently, a sequence of nonlinear operations is executed in order to optimize network performance.

CNNs process the parameters of the previous layer of neurons through convolutional operations to simplify the network structure. The process is formulated as follows:

1 $$x_{i}^{n} = f(\sum\limits_{{j \in M_{i} }} {x_{j}^{n - 1} * k_{ij}^{n} + b_{i}^{n} } )$$

where xin is the matrix of the i-th feature map of the nth layer; f(x) is the activation function and Mi is the combination of feature maps; * is the convolution operation; kijn is the convolution kernel matrix; and bin is the bias.

A pooling layer can be added after the convolution layer to downscale the feature map that is created after convolution, which can speed up the calculation and reduce the number of parameters. The following is the calculation formula for the pooling operation:

2 $$x_{i}^{n} = f({\text{down(}}x_{i}^{n - 1} ))$$

where xin is the amount of features before pooling; xin-1 is the amount of features after pooling; down(x) is the pooling function.

The fully connected layer is a commonly used layer in neural networks, which is used to spread the features learned in the previous layers into a one-dimensional feature vector, and the formula for this process is as follows:

3 $$x^{{\text{n}}} = f(w^{{\text{n}}} x^{{{\text{n}} - 1}} + b^{{\text{n}}} )$$

Where w is the weight matrix of the network and b is the bias.

The cross-entropy loss function serves as the goal function in this work. A popular classification loss function for calculating the discrepancy between the true label and the model prediction is the cross-entropy loss function. The following is its formula for calculation:

4 $$L = - \sum\nolimits_{i = 1}^{N} {y_{i} \log \left( {p_{i} } \right)}$$

Where L denotes the cross-entropy loss, N denotes the number of categories, yi denotes the one-hot coding of the true label, and pi denotes the prediction probability of the model for the ith category. By minimizing the cross-entropy loss, the evaluation value of the model can be made closer to the real label, thus improving the classification performance of the model.

#### Adam optimizer

The Adam optimizer is an adjustable learning rate optimization technique that addresses the issue of too large or too small learning rates that conventional gradient descent methods may encounter in deep learning training^37^. It combines the benefits of the AdaGrad and RMSprop algorithms^38^. By integrating the first-order moment estimate (the mean) with the second-order moment estimation (the variance), the Adam method adaptively modifies the learning rate.

In order to achieve adaptive tuning of the various parameters, it specifically computes the adaptive learning rate for each parameter using the first-order moment estimate (i.e., the exponential moving average of the gradient) and the second-order moment estimate (i.e., the exponential moving average of the gradient squared) of the historical gradient.Faster convergence, insensitivity to hyper-parameter selection, and adaptive optimization of the learning rate for big datasets and high-dimensional parameter spaces are some benefits of Adam's approach.

#### Optimized 1D convolutional neural network construction

**Figure 1:**
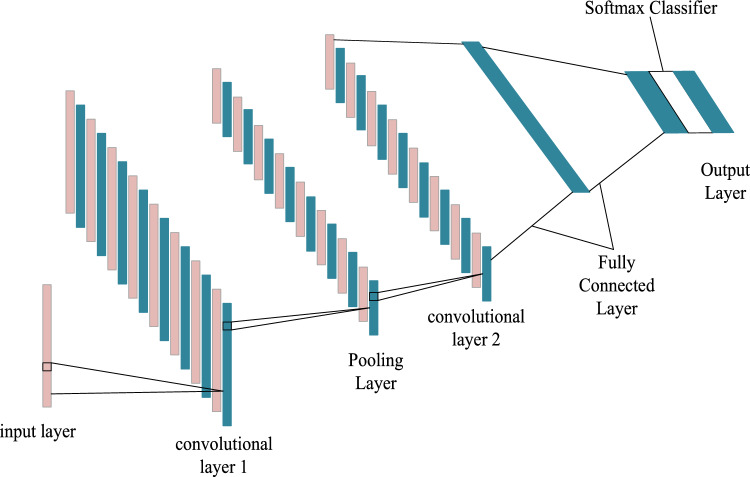
Optimized 1D-CNN structure.

The Optimized 1D-CNN structure is used in this study, and its structure is shown in Fig. 1.

The connections between the layers are forward propagation and backward propagation. Compared with the standard CNN model, the optimization scheme of the 1D-CNN model is as follows.

1. An extra convolutional layer is added and placed before the fully connected layer for deeper feature extraction.
2. The original Sigmoid function is replaced with a ReLU function, a change that effectively mitigates the problems of gradient explosion and gradient vanishing.
3. In order to prevent overfitting in the network training process, the dropout technique is introduced into the 1D-CNN model, which makes some neurons stop working during the forward propagation of the convolutional neural network to reduce the amount of redundancy.
4. Use Adam optimizer to calculate and update the network parameters of 1D-CNN model. With the diagonal scaling property of the gradient of Adam algorithm, a large number of datasets or parameters are solved to deal with the nonlinear relationship of slope features.

The literature claims that CNNs are highly sensitive to hyperparameters and that using various hyperparameters can result in significantly different model performances even when the CNN structure remains the same^39^. Thus, in order to enhance the performance of CNN models, further research must be done on the application of automatic optimization and hyperparameter adjustment techniques.

## Bayesian optimization CNN hyperparameter (B-1D CNN) based algorithm

By continually building a Gaussian process model to estimate the posterior distribution of the function and using that distribution to choose the next sampling point, the Bayesian optimization approach progressively optimizes a function for black-box functions^40^. The prior distribution selection, cost function definition, sampling technique selection, and Gaussian process model update are the main steps in the algorithm. In machine learning model tuning and neural network structure search, Bayesian optimization is commonly utilized because it is more efficient and can identify the global best solution with fewer samples than classic algorithms like grid search and random search.

The detailed iterative process of Bayesian optimization framework is shown in Table 1, where is the set of xn neural network hyperparameters, the range of values is limited by the input space of χ; yn is the neural network observation corresponding to xn, and εn is the observation error. α is the acquisition function, we can maximize the acquisition function to get the search value of x in the next step; D1:n is the set of observations, which can be expressed as .D1:n = {(× 1,y1) ∪ (× 2,y2) ∪ …(xn,yn)}.

**Table 1:** Bayesian optimization framework.

Bayesian optimization framework
1: For n = 1,2,…do
2: Maximize the collection function to get the next evaluation point:$x_{{\text{n}}} = \arg \max_{{{\text{x}} \in \chi }} \alpha \left( {x|D_{{1:{\text{n - 1}}}} } \right)$
3: Bring the selected evaluation point into the objective function $y_{n} = f\left( {x_{n} } \right) + \varepsilon_{n}$ to get the observation value
4: Integrate the data:$D_{1:n} = D_{1:n - 1} \cup \left\{ {{\text{x}}_{n} ,y_{n} } \right\}$ and update the probabilistic proxy model
5: End for

### Open pit mine slope stability evaluation based on Bayesian optimized CNNs

**Figure 2:**
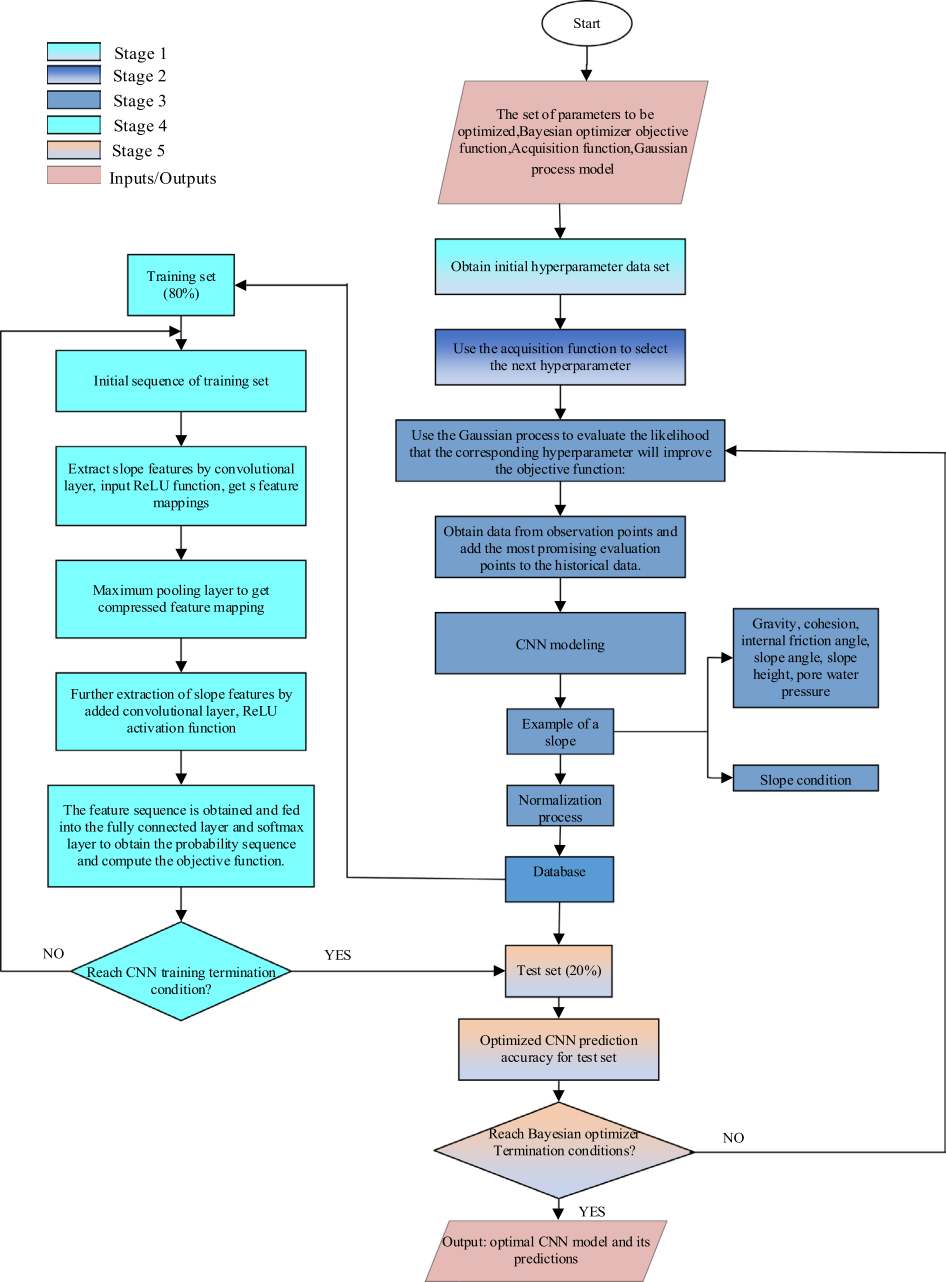
Slope stability prediction model based on Bayesian optimization CNN.

The procedure for evaluating the convolutional neural network (CNN) model's slope stability using a Bayesian optimizer is shown in Fig. 2. First, we establish the CNN model's hyperparameters using the Bayesian optimizer. Next, a training set and a test set are created from the slope stability data. We feed the training set's data into the initialized CNN model, run operations on it like convolution and pooling, then use the fully connected and Softmax layers to produce probability sequences.

The objective function is then calculated. If the termination condition is not met, the convolution kernel and bias matrix are updated using Adam's technique, and the computation is carried out until the objective function converges or achieves the predetermined number of training times. We supplement the historical data used by the Bayesian optimizer with the hyperparameters and classification accuracy data once training is over. Subsequently, we retrain the CNN model, determine the probability of utilizing a Gaussian process to improve the objective function, and choose the subsequent set of hyperparameters using the acquisition function. Until the Bayesian optimizer achieves the termination condition, these steps are repeated. In the end, using the Bayesian optimizer to continuously refine the CNN model's hyperparameters leads to improved model performance.

## Example validation

### Selection of input parameters and creation of data set

The excavation of coal mines creates surface coal mine slopes, and a number of factors can affect how stable these slopes are. When calculating the factor of safety, six parameters are included: weight (*X*1), cohesiveness (*X*2), angle of internal friction (*X*3), slope angle (*X*4), slope height (*X*5), and pore water pressure (*X*6). These factors are also considered when modeling and analyzing slope stability. The six parameters—weight capacity (*θ*), cohesion (*C*), angle of internal friction (*φ*), slope angle (*β*), slope height (*H*), and pore water pressure (*P*)—were also taken into account, drawing from earlier research^20,41,42^. Thus, the network model's input variables were chosen from among the six metrics of weight (*X*1), cohesiveness (*X*2), angle of internal friction (*X*3), slope angle (*X*4), slope height (*X*5), and pore water pressure (*X*6).

The "Coal Industry Surface Mine Slope Engineering Design Standard" (GB51289-2018) states that the slope stability state (S) is classified into four categories (Table 2 Variable Definition Values) and that these values are used as the neural network model's output. The four categories in which the slope stability state (S) is classified are unstable, unstable, basically stable, and stable, and they correspond to the output target values of 0, 1, 2, and 3.

**Table 2:** Variable value definitions.

Variable name	Type	Designator	Classification basis	Target value
Slope stability *S*	Instability	I	*F*_*S*_ < 1.00	0
	Not stable	II	1.00 ≤ *F*_*S*_ < 1.05	1
	Basic stability	III	1.05 ≤ *F*_*S*_ < 1.2	2
	Stable	IV	*F*_*S*_ ≥ 1.2	3

**Figure 3:**
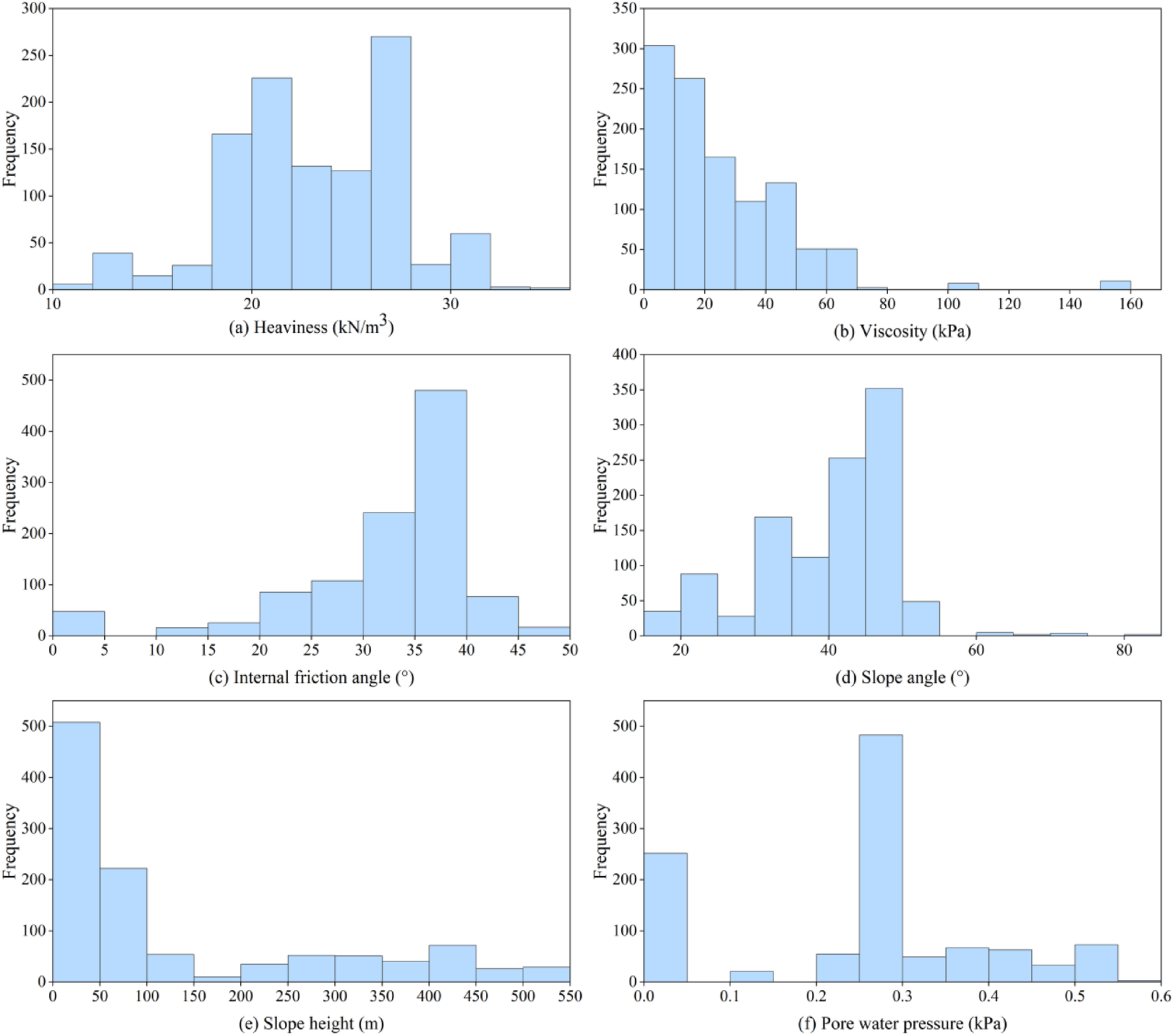
The histograms of the input variables.

This paper builds a dataset and gathers a large number of representative samples of slope stability influencing factors through literature reviews^43–49^ and field investigations. The types of slopes included in this dataset are rocky and dump slopes. The purpose of this is to confirm the viability and validity of the model. Within the 1099 slope examples in the used dataset, 276 come from stabilized slopes, 275 from unstable slopes, and 548 from understabilized, or essentially stabilized, slopes. Among the slope types are rocky slopes (555) and drainfield slopes (544). Figure 3 displays the input variable histograms. As can be seen, a variety of predictor factors are included in the data used. It is important to note that Fig. 3 indicates that the study's conclusions are more trustworthy when the range of data points is more condensed.

**Figure 4:**
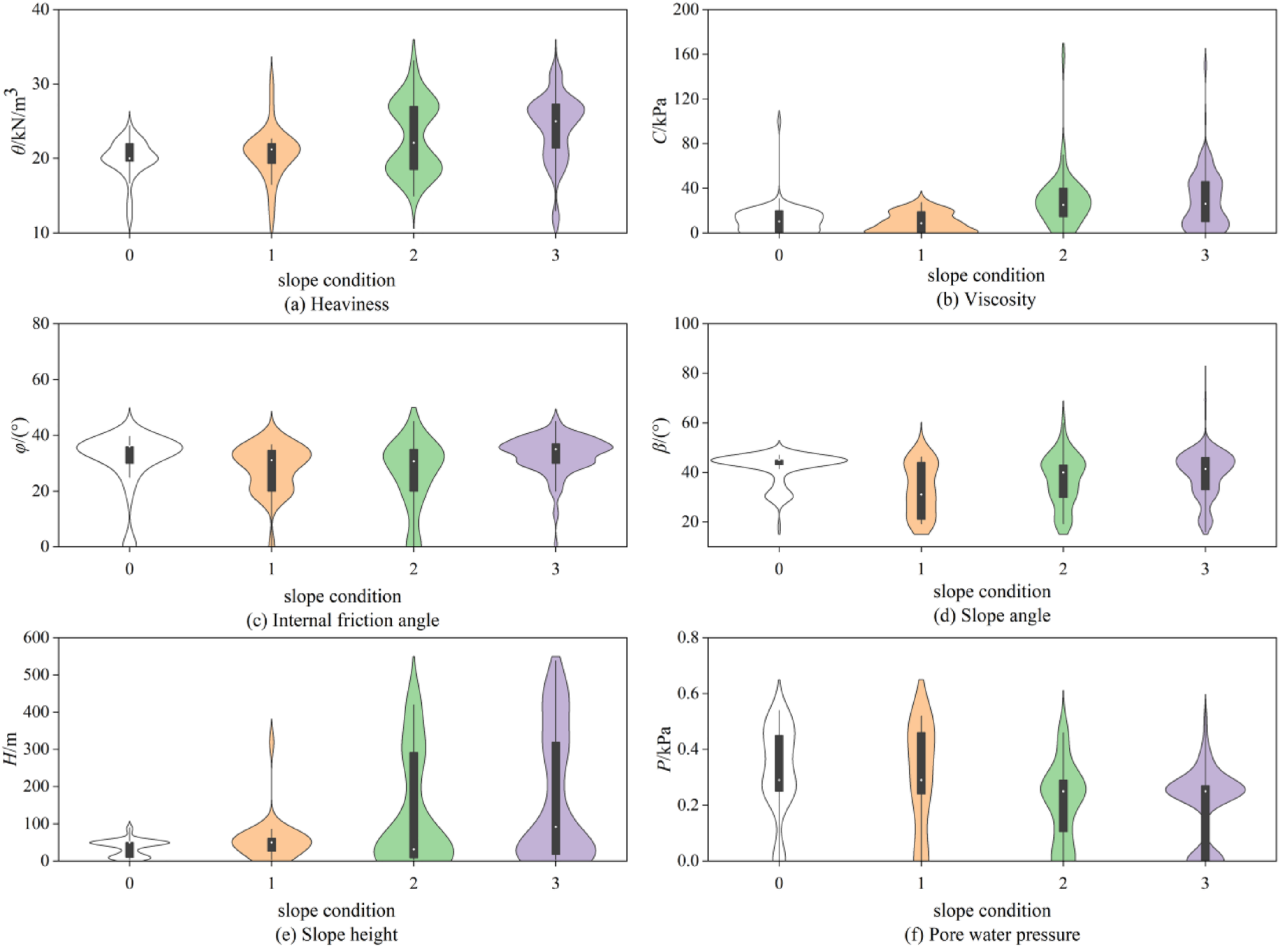
Violin Diagrams of Distribution of Characteristic Parameters from Slope in Data Set.

The distribution of the 1099 distinctive parameters of the collected slopes is shown in Fig. 4, which is a violin diagram. The violin diagram's outer contour width indicates the density of the distribution of the data points; the box diagram is represented by the rectangle in the center of the diagram; the upper and lower quartiles are represented by the diagram's edges, respectively; and the median is located in the middle. The distribution of gravity is primarily within the range of 10 ~ 30kN/m3, cohesion is primarily between 0 and 40 kPa, internal friction angle is primarily between 20 and 40°, and slope angle is primarily between 20 and 50°, as can be seen in the figure. The bulk of the slopes in the unstable and unstable conditions are 50 ~ 500 m; the height of the slope in the stable and essentially stable state is 0 ~ 100 m. The range of 0.2 ~ 0.2 m is where the majority of the pore water pressure is distributed. The major distribution of pore water pressure is between 0.2 and 0.6 kPa, and its density of distribution is higher than that of the unstable condition.

The total dataset was randomly split into two halves in order to validate the new model: the training dataset (876 data points) and the test dataset (219 data points). The remaining 20% of the data points were utilized to verify the produced model's prediction capacity. Table 3 displays the range of input variables for the training and test datasets. For every predictor variable, the table displays the minimum (Min), maximum (Max), mean (Mean), and standard deviation (Std).

**Table 3:** The ranges of predictive variables for training and testing datasets.

Parameter	Dataset	Min	Max	Mean	Std
θ (kN/m^3^)	Training	11.82	35	25.51	4.82
	Testing	11.94	35	25.32	4.21
C (kPa)	Training	0	159	30.22	24.81
	Testing	0	150	30.12	23.46
φ (°)	Training	0	40.1	31.68	8.75
	Testing	0	40	30.22	8.46
β (°)	Training	16	80	39.31	9.38
	Testing	16	78	38.23	9.23
H (m)	Training	3.59	538	138.17	159.84
	Testing	3.49	538	137.21	157.63
P (kPa)	Training	0	0.56	0.23	0.15
	Testing	0	0.56	0.22	0.14

Table 4 displays a portion of the slope case data in order to visually represent the data that was collected (just a portion of the samples are displayed owing to space limits).

**Table 4:** Influencing factors and outputs of the slope stability analysis model.

No	Heaviness/(kN/m^3^)	Viscosity/kPa	Internal friction angle/(°)	Slope angle/(°)	Slope height/m	Pore water pressure/KPa	Grade
1	20.00	32.85	11.22	16.64	43.87	0.21	3
2	19.24	27.66	15.15	35.70	8.56	0.00	2
3	17.90	14.79	26.25	19.40	29.59	0.00	3
4	18.09	59.95	21.00	19.20	29.59	0.00	3
5	21.08	7.15	29.70	32.24	74.51	0.38	0
6	28.44	28.54	33.60	34.65	101.00	0.00	3
7	14.56	11.37	26.52	30.00	89.76	0.45	0
8	28.72	40.80	39.90	33.60	100.00	0.00	3
9	19.80	20.20	36.36	45.45	48.50	0.25	0
10	14.42	11.61	25.74	31.20	86.24	0.00	1

The python programming language used in the environment of this experiment, the framework is Keras version 2.11.0 and Python version 3.8.

**Figure 5:**
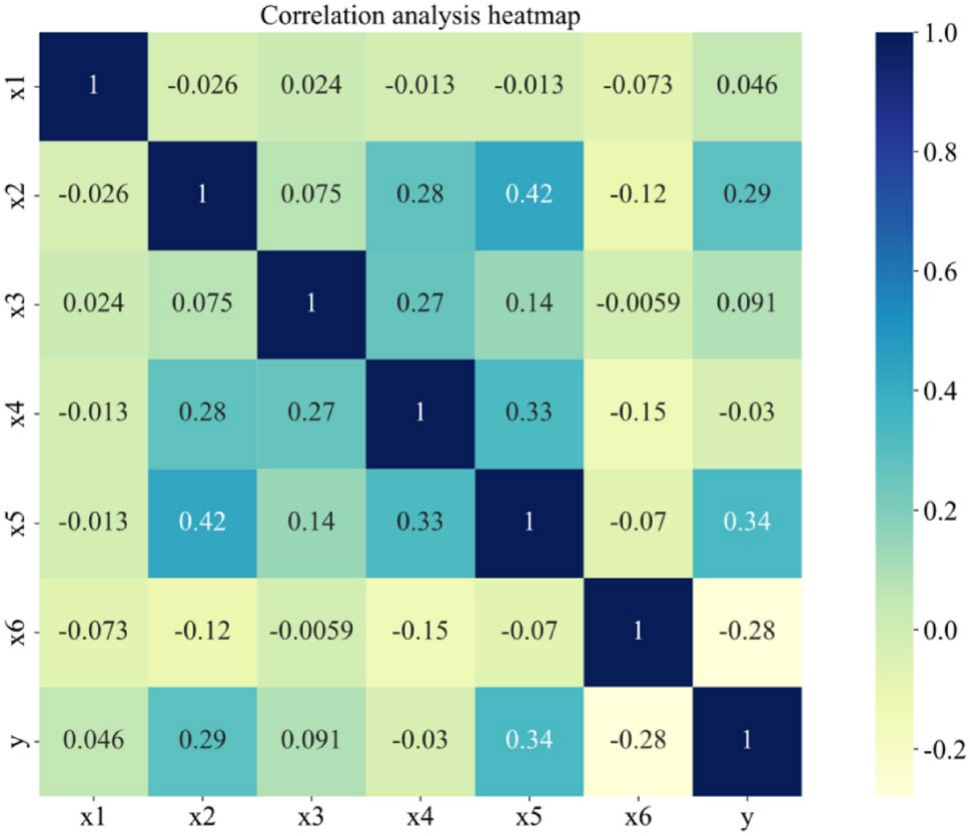
Correlation matrix heat map.

A heat map of the correlation matrix was created after using Pearson's correlation coefficient to examine the relationship between the influences in the dataset and the output (y), as seen in Fig. 5. It is evident from the heat map that there is little association between the chosen influencing factors and the output outcomes, since all correlation coefficients are less than 0.50. It also suggests that their relationship is nonlinear and more intricate.

Due to the significant scale variances in the numerical attributes of the dataset, the pore water pressure and slope height attributes do not have the same order of magnitude.

In order to better compare, analyze, and process data, normalization is a popular data preprocessing technique that brings data with varying scales and units into the same scale range. The data set is standardized to bring uniformity to each parameter's requirements.

Standard deviation normalization is selected as the transformation function in order to maintain the original data information and distribution pattern without altering the distribution pattern of the data:

5 $$x* = \frac{x - \mu }{\sigma }$$

Where μ is the mean of all sample data, σ is the standard deviation of all sample data.

### Bayesian optimization of CNN model hyperparameters

Following the normalization of the dataset, the 1D.CNN model was trained on 80% of the data and tested on 20% of the data; this ratio of training to validation sets was found to be useful in avoiding overfitting. The following is the procedure for feeding the dataset into the CNN model and utilizing a Bayesian optimizer to optimize the hyperparameters: What 1D-CNN hyperparameters need to be optimized is listed in Table 5: The hyperparameters that will be automatically changed during the Bayesian optimization process to optimize the CNN model's performance are the number of convolutional kernels (Filters), convolutional kernel size (Kernel-size), maximum pooling-window size (Pooling-size), number of neurons in the fully-connected layer (Units), batch size (Batch-size), and number of training repetitions (Epochs).Based on the findings of earlier studies, each hyperparameter's search range is determined. The prior distributions of the hyperparameters were selected based on statistical prior knowledge to guarantee that the search space included all hypothetical possibilities and was hence comprehensive,the range of every hyperparameter was subsequently determined by numerous iterative trials and errors^50,51^.

**Table 5:** Hyperparameter search optimization settings.

Variant	Hyperparame	Search space
Y1	Filters	(2.0,128.0)
Y2	Kernel-size	(1.0,5.0)
Y3	Pooling-size	(1.0,2.0)
Y4	Units	(31.0,100.0)
Y5	Epoch	(50.0,300.0)

The test results are categorized into four types according to the combination of the actual situation and the evaluation results: True Stable (*TP*), False Destruction (*FN*), False Stability (*FP*) and True Destruction (*TN*), and the total number of test samples *N* = *TP* + *FN* + *FP* + *TN*, and the confusion matrix of the classification results is shown in Table 6.

**Table 6:** Confusion matrix of classification results.

Actual	Evaluation results	
	Stabilization (*P*)	Destruction (*N*)
Stabilization (*P*)	True stable (*TP*)	False destruction (*FN*)
Destruction (*N*)	False stability (*FP*)	True destruction (*TN*)

Three categories of performance indicators were employed to analyze the built model in order to determine its performance. These metrics consist of the following: A. accuracy, B. precision, and C. F1-score, which is the result of the mean of precision and recall that has been reconciled. These measures aid in assessing how well the model performs on the categorization task.

6 $$A = \frac{STP + STN}{N}$$

7 $$B = \frac{STP}{{STP + SFP}}$$

8 $$R = \frac{STP}{{STP + SFN}}$$

9 $$F1 - Score = \frac{2 \times R \times B}{{R + B}}$$

Where: *S*_*TP*_ is the number of samples with confusion matrix categorized as *TP*; *S*_*TN*_ is the number of samples with confusion matrix categorized as *TN*; *S*_*FN*_ is the number of samples with confusion matrix categorized as *FN*; and *S*_*FP*_ is the number of samples with confusion matrix categorized as *FP.*

While the other indicators show the model's capacity to classify samples from various categories, Indicator A represents the model's overall performance in classification. The greater the value of these metrics, the more broadly the model can be used. The metrics show the model's overall performance in classification as well as its capacity to categorize samples from various categories; the higher the value, the better the model's performance on data that has not yet been seen. Macro-averaging, or calculating the average of the metrics for each category to represent the entire category, is utilized here to represent the other categories because it entails the thorough review of numerous categories.

**Figure 6:**
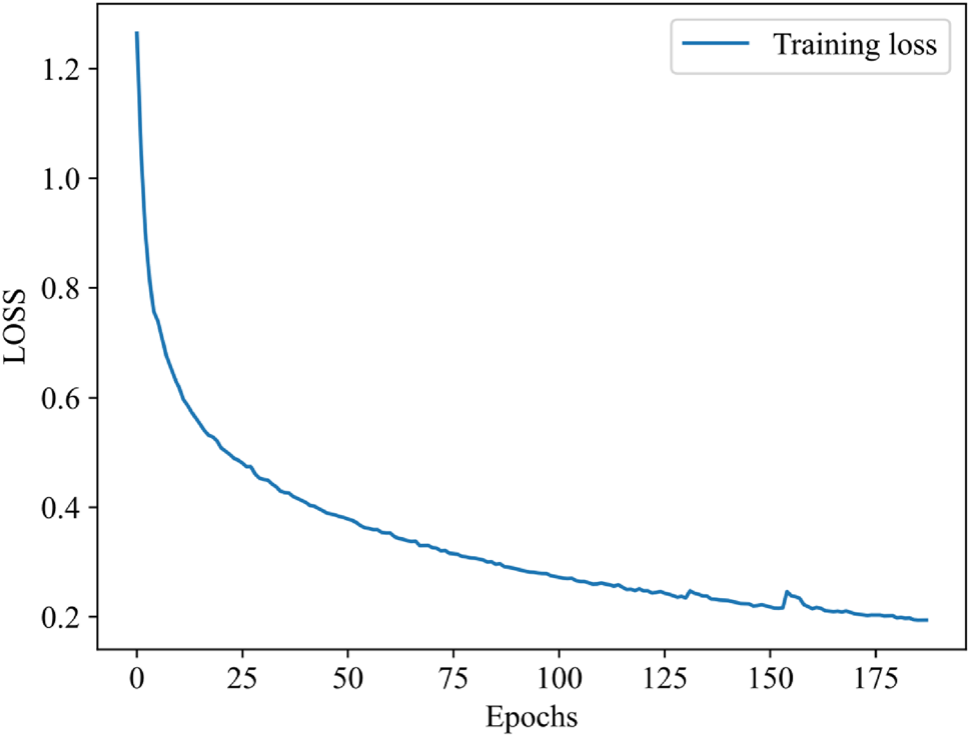
Loss curve during training.

The variation of the cross-entropy loss function (LOSS) with the number of rounds (Epoch) during training is shown in Fig. 6.

The convolutional neural network model begins to stabilize at a learning rate of 0.0001, as the figure shows, after around 175 iterations. The loss function value drops from 1.36 to 0.2, indicating a good degree of convergence of the network model.

#### Bayesian optimisation of CNN model hyperparameters

**Figure 7:**
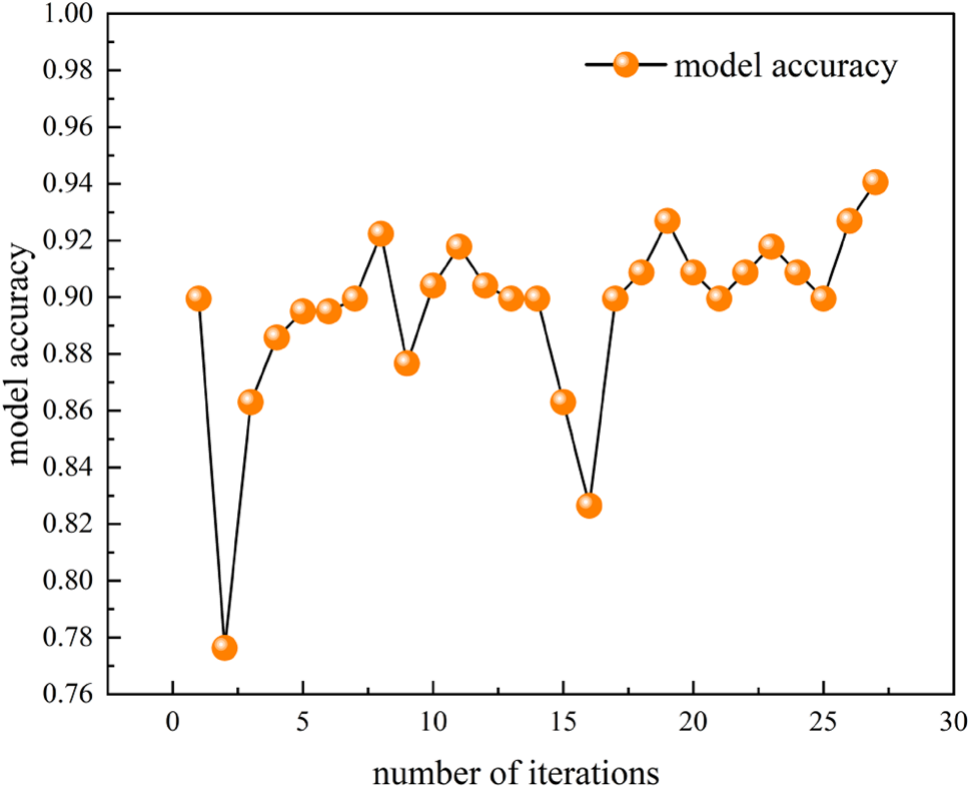
Hyperparametric sensitivity analysis.

According to Fig. 7 in the hyperparameter sensitivity analysis, it can be seen that the better hyperparameter combination was obtained at the 8th iteration, while the optimal hyperparameter combination was obtained at the 27th iteration.

According to the hyperparameter optimization results in Table 7, the optimal hyperparameter combination was obtained at the 27th iteration as follows: the CNN network depth is 1, the number of loops is 183, the number of convolutional kernels is 40, the convolutional kernel size is 4, the maximum pooling window is 1, and the number of neurons in the fully connected layer is 45.In order to determine the best combination of hyperparameters and enhance the CNN model's performance, the Bayesian optimizer looks for the best combination within the specified hyper-parameter value interval.

**Table 7:** Hyperparameter optimization results.

Number of iterations	Objective function	Number of cycles	Number of convolution kernels	Convolution kernel size	Maximum pooling window	Amount of neurons in fully connected layers	Result evaluation
1	0.8995	106.2	121.8	3.928	1.599	41.77	Accept
2	0.7763	73.4	9.319	4.465	1.601	79.86	Accept
3	0.863	53.09	124.2	4.33	1.212	43.55	Accept
4	0.8858	77.51	40.33	3.099	1.432	51.09	Accept
5	0.895	141.8	19.58	2.169	1.366	62.47	Accept
6	0.895	167.8	27.16	3.057	1.592	34.21	Accept
7	0.8995	141.1	23.49	1.26	1.949	97.63	Accept
8	0.9224	171.3	40.38	1.391	1.684	61.37	–
9	0.8767	68.31	64.39	1.138	1.909	48.86	Accept
10	0.9041	149.4	41.28	3.08	1.547	43.75	Accept
11	0.9178	172.4	87.85	1.37	2.0	100.0	Accept
12	0.9041	149.7	43.52	2.75	1.95	44.8	Accept
13	0.8995	156.0	64.55	1.0	2.0	54.35	Accept
14	0.8995	154.4	41.53	1.0	2.0	59.81	Accept
15	0.863	146.7	50.92	4.287	1.005	38.31	Accept
16	0.8265	161.3	48.81	1.333	2.0	39.34	Accept
17	0.8995	140.5	48.94	1.951	2.0	46.5	Accept
18	0.9087	179.9	39.47	3.495	1.468	59.35	Accept
19	0.9269	176.6	37.19	4.032	1.392	68.73	Accept
20	0.9087	179.1	50.1	4.306	1.077	66.79	Accept
21	0.8995	188.7	42.67	1.0	2.0	69.36	Accept
22	0.9087	171.1	46.18	3.966	1.489	75.64	Accept
23	0.9178	180.6	25.65	1.829	1.829	60.69	Accept
24	0.9087	177.4	51.91	2.899	1.542	56.3	Accept
25	0.8995	184.8	63.6	5.0	1.0	61.51	Accept
26	0.9269	190.0	54.16	4.022	1.03	49.25	Accept
27	0.9406	183.7	40.79	4.868	1.146	45.96	Best

### Comparison experiment

**Figure 8:**
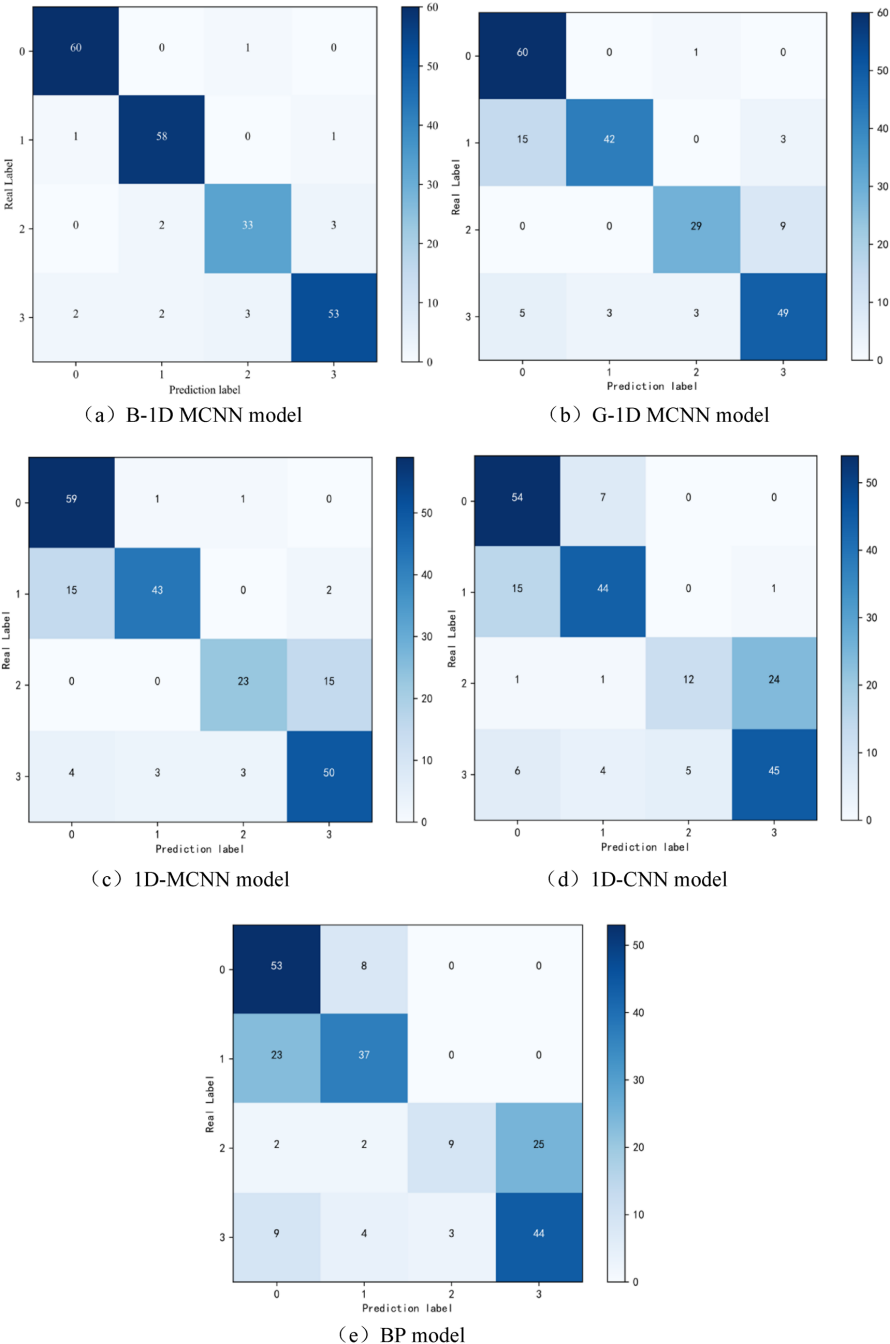
Confusion matrix for different models.

The purpose of this paper is to assess the efficacy of the approach presented in this paper for the stability analysis of slopes and the model's classification performance. To this end, a comparison experiment was designed using the same dataset in the Bayesian optimization of the 1D-MCNN model (B-1D MCNN), the optimized 1D-CNN model by genetic algorithm (G-1D MCNN), the optimized CNN model (1D-MCNN), the standard CNN model (1D-CNN), and the BP neural network model (BP) on the test set to diagnose the accuracy. The optimized methods in the 1D-MCNN model all employ the dropout technique to remove some of the hidden layer neurons and update the network's parameters using the Adam optimizer.This paper employs the F1-score, precision, and accuracy as indicators and statistically the confusion matrix of the test data of several models to quantitatively evaluate the efficacy of various statistical modeling methods for stability analysis of slope data. The specific results are shown in Fig. 8, according to Fig. (a).

The categories of predictions are represented by each column of the confusion matrix, and the total number of each column shows how many predictions the model made overall for that category. For instance, the first column's total number of 63 signifies that the model's total number of predictions for category 0 is 63. The total for each row indicates the number of data instances that truly belong to that category, while each row itself shows the actual category. When the first row's total is 61, for instance, it means that 61 data instances truly fall under category 0. The confusion matrix shows each specified value as the number of actual data points that the model expected to fall into a certain category. In the first row, for instance, a value of 60 in the first column means that 60 data instances are correctly predicted as category 0, even though they actually belong to category 0. According to this, one case that truly belongs to category 0 but was mistakenly forecast to be category 2 is indicated by a value of 1 in the first row's third column.

According to Fig. 8a, among the 219 slopes to be tested, the number of slopes with correctly categorized slope data are 204, 180, 175, 155 and 143 for B-1D MCNN, G-1D MCNN, 1D-MCNN, 1D-CNN and BP, respectively.

## Discussion and generalizability validation

### Precision performance

Six fundamental characteristics are used as input indicators by the 1D-CNN model based on Bayesian optimization presented in this paper: slope height, slope angle, capacity, cohesion, angle of internal friction, and pore water pressure. The CNN model employs the ReLU activation function to lessen gradient vanishing and adds an additional convolutional layer for the purpose of extracting deep features. The model's capacity to learn features is optimized when the dropout approach and Adam optimizer are combined. The network depth and learning rate of the enhanced CNN are modified using Bayesian optimization, taking into account the impact of hyperparameters on slope stability assessment. Lastly, the enhanced CNN model is fed the slope data, and the Bayesian optimizer is used to tweak the hyperparameters. With the goal of offering a dependable foundation for the design and administration of slope engineering in open-pit mines, this all-encompassing methodology integrates knowledge of slope engineering.

To assess the developed model even further, an experiment is set up in 3.3 that also compares its performance with the four fundamental neural network computational methods developed by the authors of previous research^50–52^. This is done using the same dataset on the Bayesian optimization of the optimized 1D-CNN model, the 1D-CNN model optimized by the Genetic Algorithm, the optimized 1D-CNN model, the standard 1D-CNN model, and the BP neural network model on the test set to determine the accuracy. The optimized methods in the first three models in this paper use the dropout technique to remove some neurons in the hidden layer and update the network's parameters using the Adam optimizer.

In reference to additional model parameters, such as the number of layers in the hidden layer, the number of convolutional kernels, the size of the convolutional kernel, the maximum pooling window, the number of neurons in the fully connected layer of the decision size, and so on, the procedure involves combining empirical and trial-and-error methods; first, empirical formulas are used to estimate the precise number; based on this, the trial-and-error method is used to determine the final number of the hidden layer nodes, and the final hidden is determined by comparing the model's error under the various numbers of layer nodes. Model performance and convergence speed are directly impacted by the model's learning rate; the higher the learning rate, the faster the model will converge. However, during the final stages of model training, there will be significant fluctuations that will cause oscillations and even cause the loss function value to hover around the minimum value, indicating that an optimal solution may not be found. To ensure that the model is closer to the ideal answer in the later stage, this research uses the learning rate decay strategy, starting with a bigger learning rate (n = 0.1) and decreasing the rate to 0.95n as the number of iterations increases.

**Figure 9:**
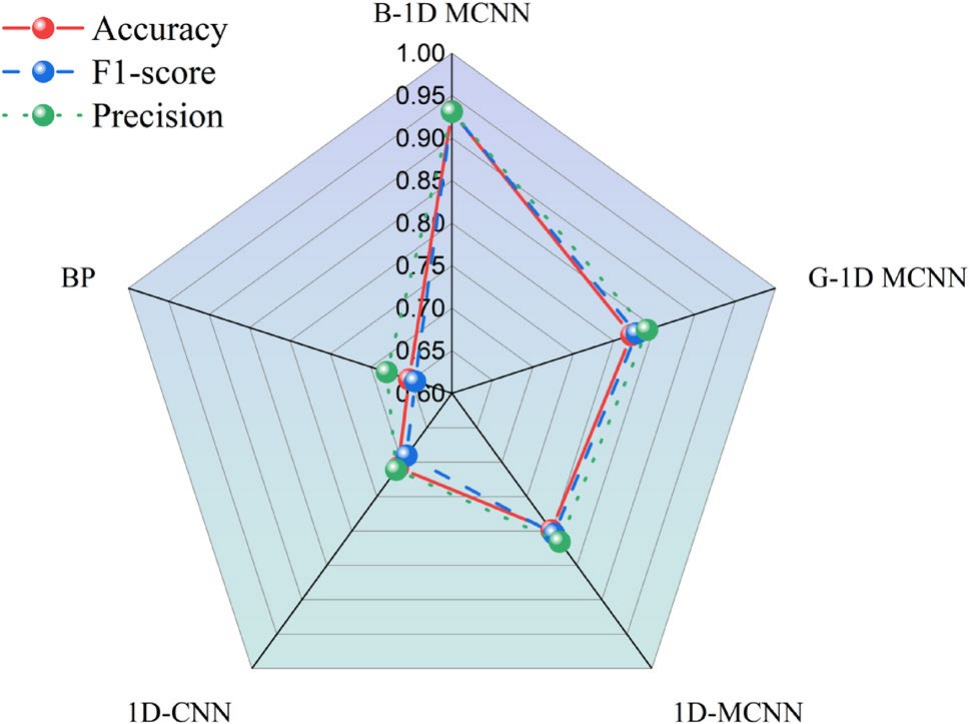
Test results of different models.

Figure 9 displays each model's overall accuracy, precision (P), and F-score statistical metrics.With an average accuracy of 79.91 percent, the optimized 1D-CNN model outperforms the ordinary 1D-CNN model by 9.13 percent, making it the more accurate model overall. The Bayesian optimization of hyperparameters can improve the model classification performance, so it is necessary to optimize them. The F1-score of the model (B-1D MCNN) is 93.10%, which is improved by 10.26% and 12.67% compared with the 1D-CNN model optimized by the genetic algorithm and the improved CNN model, respectively; the precision of the model (B-1D MCNN) is 93.11%, which is improved by 8.98% compared with the G-1D MCNN.

### Data-driven decision making and physical interpretation of model prediction results

In the slope stability prediction model, the input parameters can be regarded as a feature map, each pixel in the image contains the corresponding slope stability influence factor, and each grid cell of the input parameters is regarded as a one-dimensional vector. The input data reaches the convolutional layer after passing through the input layer, which consists of multiple convolutional kernels, similar to the neurons of a feed-forward neural network, and the function of the convolutional layer is to carry out feature extraction after convolutional operation of the input data. The pooling layer is located behind the convolutional layer and is responsible for feature selection and information filtering of the feature map extracted by the convolutional layer, splitting it into smaller blocks, and extracting the corresponding features in each corresponding block. Pooling operation can accelerate the network computation speed, avoid overfitting and other situations in the process of computation, and improve the robustness of the network model. The fully connected layer is located in the last part of the implicit layer of the convolutional neural network and receives the pooled feature map, which loses its spatial topology in the fully connected layer and is expanded into vectors and classified by the activation function. The use of the activation function can enhance the nonlinear expression ability of the network, thus improving the generalization of the network structure, the network after a series of operations, and finally the slope stability state prediction information will be conveyed to the output layer for output.

Among the many factors affecting slope stability are stratigraphy and lithology, geologic formations and ground stress, geotechnical structure, water action, and slope geometry and surface morphology.In general, the formula for calculating the factor of safety commonly used in slope stability analysis can be expressed in the following form:

$${\text{Factorof Safety }} = {\text{ Shear Strength }}/{\text{ Shear Stress}}.$$

In particular, the shear strength is usually calculated based on the cohesion and the angle of internal friction, while the shear stress is determined by the slope forces and groundwater conditions.

Specifically, the relationship with other factors factors is as follows:

Heaviness (*X*1): Heaviness can affect the stress distribution in the rock mass and thus the stability of the rock mass. Usually, the gravity affects the effective stress of the rock mass and thus the calculation of shear strength.

Cohesion (*X*2) and angle of internal friction (*X*3): Cohesion and angle of internal friction are mechanical parameters of the rock mass, they directly affect the shear strength of the rock mass, and they are important input parameters in the calculation of the factor of safety.

Slope Angle (*X*4) and Slope Height (*X*5): The slope angle and slope height of the slope will directly affect the force condition of the slope, which in turn will affect the calculation of shear stress. In general, steeper slope angles and higher slope heights result in larger shear stresses and require a greater factor of safety to ensure slope stability.

Pore water pressure (*X*6): Pore water pressure affects the effective stresses in the rock mass, which in turn affects the calculation of shear strength. A larger pore water pressure reduces the effective stress in the rock mass, resulting in a lower stability of the slope, and a larger safety factor is needed to compensate for this effect.

Therefore, six indicators, namely, gravity (*X*1), cohesion (*X*2), angle of internal friction (*X*3), slope angle (*X*4), slope height (*X*5), and pore water pressure (*X*6), are selected as input variables for the network model.

### Statistical error analysis

Based on the "Coal Industry Surface Mine Slope Engineering Design Standard" (GB51289-2018) (Table 8), combined with the actual slope dataset, the value of the safety coefficient is set to 1.2, and the slope status is classified into four categories according to the range of the safety coefficient, as shown in Table 2 above.

**Table 8:** Selection of slope safety factor *F*_s_.

Slope type	Years of service (a)	Safety factor FS
Where there is a building of particular importance on the slope or where a landslide would cause significant loss of life or property	> 20	> 1.5
Final slopes of extraction sites	> 20	1.3 ~ 1.5
Non-working gang slopes	< 10	1.1 ~ 1.2
	10 ~ 20	1.2 ~ 1.3
	> 20	1.3 ~ 1.5
Working gang slopes	Temporarily	1.0 ~ 1.2
Outer Drainage Slope	> 20	1.2 ~ 1.5
Inner Drainage Slope	< 10	1.2
	≥ 10	1.3

**Figure 10:**
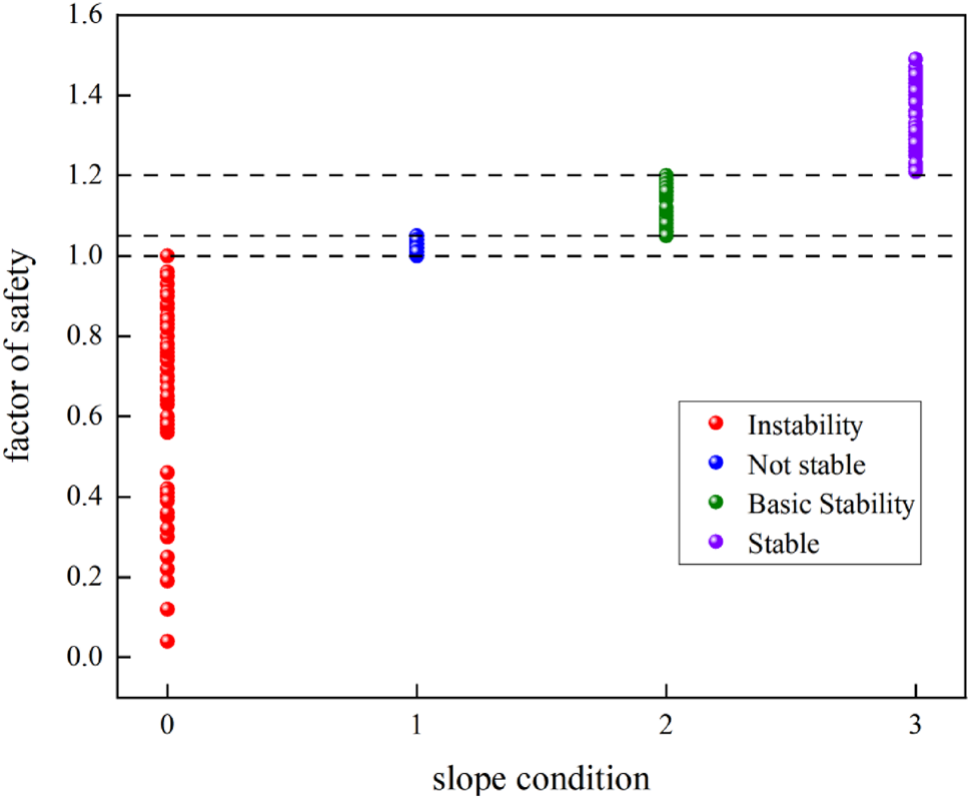
Test set slope safety factor.

Where the test set of true slope states 0 (61), 1 (60), 2 (38), 3 (60),the specific range of test set slope safety factors is shown in Fig. 10 below:

Where the correctly predicted slope states 0 (60), 1 (58), 2 (33), 3 (53), for a total number of errors of 15. As shown in Table 9 below:

**Table 9:** Test set slope condition statistics.

	Real slope state	Total number	Predicted slope condition				Number of errors	Total number of errors
			0	1	2	3		
			Total number					
Test set	0	61	60	0	1	0	1	15
	1	60	1	58	0	1	2	
	2	38	0	2	33	3	5	
	3	60	2	2	3	53	7	

**Figure 11:**
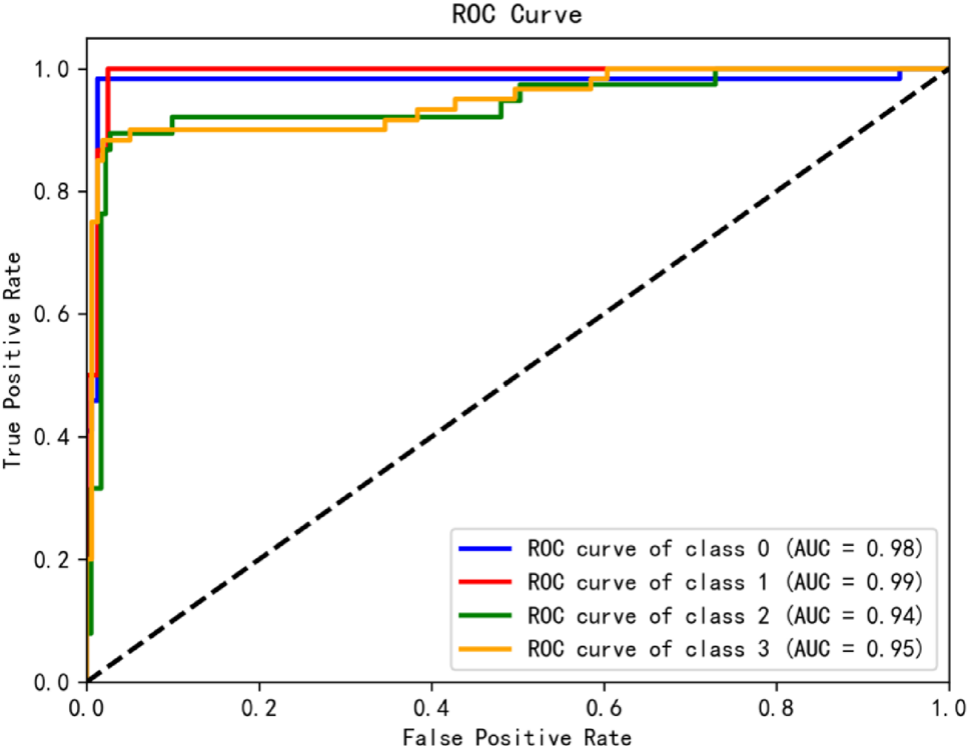
ROC curve of the model.

Figure 11 shows the ROC curve and the AUC value in the evaluation index of B-1D MCNN.The ROC curve, a graph with the false positive rate (FPR) as the x-axis and the true positive rate (TPR) as the y-axis, is usually expressed in the range of 0 to 1. The closer the ROC curve is to the point (0,1) in the upper left corner of the graph, the more the AUC value of each category is ≥ 0.94, indicating that the model has better overall performance under different thresholds and has high classification performance.

### Effect of training dataset length on the model

The performance of the trained CNN is significantly impacted by the size of the training dataset T for the majority of neural network assessment models. It is advised that T be at least 10d, where d is the number of random variables, in accordance with earlier studies^53^. The paper fixes the length of the evaluation dataset and investigates the impact of the training dataset length on the model in order to study the influence of T on the performance of the trained CNN model. The evaluation dataset was designated "50–50", "150–50", "200–50", "250–50", "300–50", "350–50", and "350–50" and its length was set to 50 groups. The CNN was trained using seven different T values: 50, 150, 200, 250, 300, 350, and 400. 50", "300–50," "350–50," and "400–50," or the length of the test set data minus the length of the training data set.

**Figure 12:**
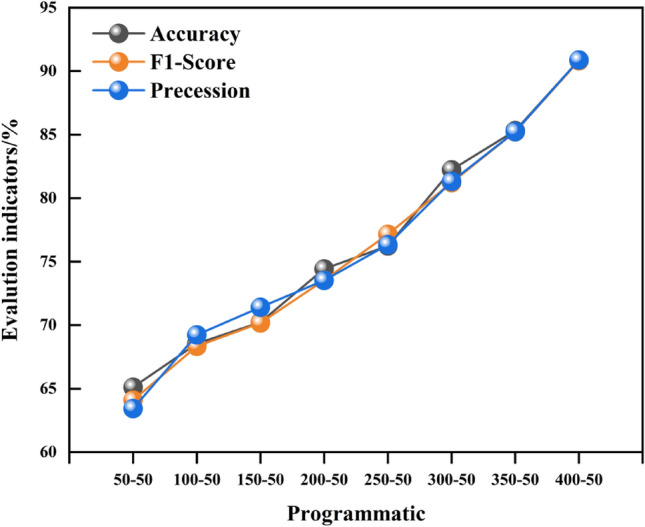
Prediction results of slope stability under different training dataset lengths.

Ten distinct and separate training datasets are created for each of the ten B-1D MCNNs at a particular T in order to prevent unpredictability in the training process. To obtain the trained B-1D MCNN model at T = 50, for instance, 50 sets of random samples and 50 values of the limit state function are randomly selected from a large number of datasets. To get 10 trained B-1D MCNNs, repeat this process ten times. These B-1DCNNs are then validated using a validation data suite consisting of V = 50. The trained B-1DCNN's performance at a specific T was assessed using the average values of A, F1-Score, and R. Figure 12 displays the average assessment findings utilizing A, F1-Score, and B evaluation.

Figure 12's results demonstrate that an increasing trend in the training dataset corresponds with an increasing trend in the model's performance. This suggests that the size of the training dataset and the model's generalization performance have a positive link. Thus, in real-world applications, it is necessary to focus on gathering additional training datasets in order to increase the model's accuracy.

### Validation of generalizability

As seen in Table 10, a new test set comprising dump and rock slopes was created based on the field dataset of a region in order to further assess the model's (B-1D MCNN) generalization capacity.

**Table 10:** Data to be evaluated.

Slope type	No	Heaviness/(kN/m^3^)	Cohesion/kPa	Internal friction angle/(°)	Slope angle/(°)	Slope height/m	Pore water pressure/KPa	Safety coefficient	Grade
Rocky slopes	1	22.40	10.00	35	45	10.00	0.40	0.90	0
	2	20.00	20.00	36	45	50.00	0.50	0.83	0
	3	20.00	0.00	36	45	50.00	0.25	0.79	0
	4	18.00	5.00	30	20	8.00	0.30	1.58	3
	5	25.0	46	35	47	443	0.25	1.28	3
	6	27.30	14	31	41	110	0.25	1.249	3
	7	18.50	25.00	0	30	6.0	-	1.09	2
Dump slopes	8	27.30	24.96	30.07	52.50	96.60	0.26	1.21	3
	9	21.28	10.30	33.60	43.20	10.00	0.40	0.86	0
	10	14.00	11.85	26.52	30.30	86.24	0.44	0.66	0
	11	20.42	11.49	19.00	21.34	11.71	0.41	1.38	3
	12	21.00	19.40	36.72	44.10	49.00	0.25	1.00	1
	13	24.25	44.16	34.30	37.05	430.55	0.26	1.32	3
	14	26.21	10.20	39.00	41.41	526,55	0.26	1.48	3
	15	18.00	24.00	30.20	45.00	20.00	0.12	1.12	2
	16	18.50	25.00	10.00	30.00	26.00	0.25	1.09	2

**Figure 13:**
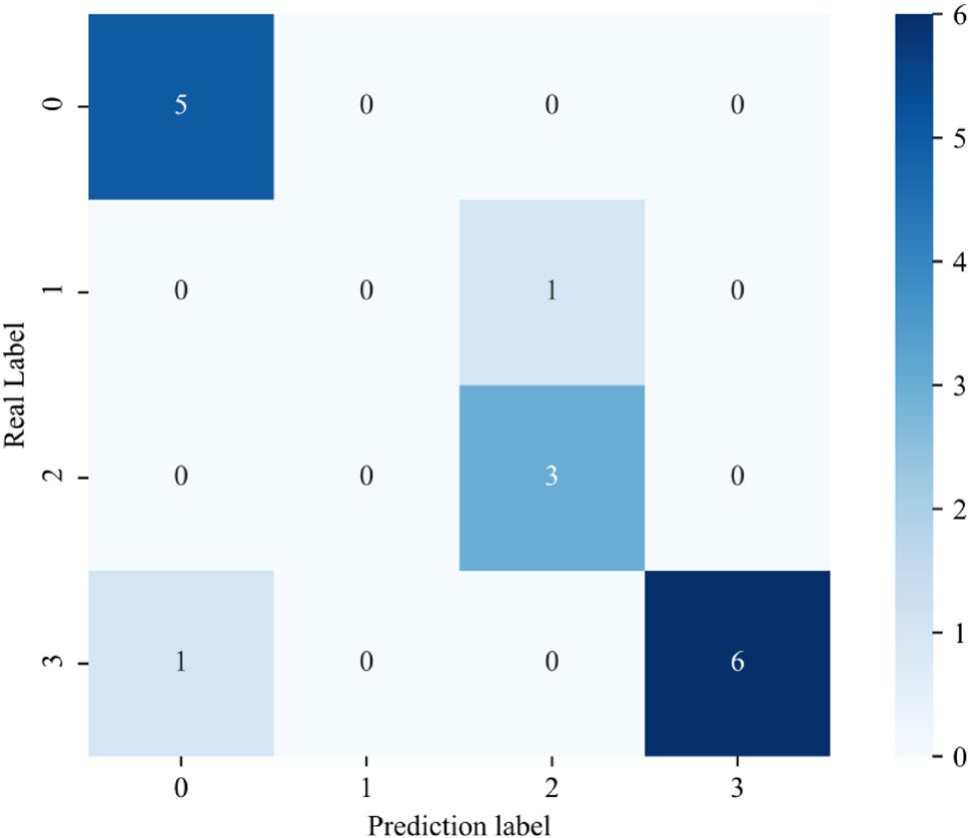
Classification results of the new test set.

The examined slope assessment indices were first extracted and standardized. Following that, the trained 1D-MCNN model receives the sample parameters, and the slope stability grade is the output. The data was fed into the B-1D MCNN model, yielding a classification accuracy of 87.5%. The test data's confusion matrix was then tallied, and the outcomes are displayed in Fig. 13. It is evident that the model maintains minimal confusion across various categories and can correctly classify and identify each category when presented with fresh test data. These outcomes provide more evidence of the model's resilience in this study and its capacity to manage challenging assignments. The accuracy rate differs slightly from the original due to the contingency and the limited number of test samples.

### Experimental limitations

The accuracy is somewhat lower because of the small, coincidental dataset that was used for the generalizability validation.

The sample data used to determine the accuracy of the current model is inadequate, and quantifying some of the parameters that affect slope stability—such as anthropogenic mining, mining circumstances, mining techniques, and management conditions—is challenging. More samples and an ongoing study will improve the accuracy of the model. It is a complicated process that depends on many variables, including the quantification of the input indicators, the rectification of the indicator data, and how to adjust to new information or modifications in mining conditions.

## Conclusion

1. Compared with the BP neural network, the optimized convolutional neural network with genetic optimization, the one-dimensional convolutional neural network, the optimized one-dimensional convolutional neural network, and the B-1D MCNN neural network model, the performance of the B-1D MCNN-based slope stability evaluation model was improved by 10.96% to 27.85%, 10.26% to 28.555%, and 8.98% to 25.5%, which comprehensively illustrates the better performance of the slope stability evaluation model based on B-1D MCNN.
2. The generalization performance of the B-1D MCNN slope stability evaluation model is 87.5%, indicating that the model can evaluate slope stability well; the improvement of the B-1D MCNN slope stability evaluation performance with the increase of the length of the training dataset indicates.

## Data Availability

The datasets used and/or analysed during the current study available from the corresponding author on reasonable request.
